# Structure and Transport Properties of Mixed-Matrix Membranes Based on Polyimides with ZrO_2_ Nanostars

**DOI:** 10.3390/polym8110403

**Published:** 2016-11-18

**Authors:** Maria P. Sokolova, Michael A. Smirnov, Pavel Geydt, Alexander N. Bugrov, Sami-Seppo Ovaska, Erkki Lahderanta, Alexander M. Toikka

**Affiliations:** 1 Saint Petersburg State University, Department of Chemical Thermodynamics & Kinetics, Universitetsky pr. 26, Peterhof, Saint Petersburg 198504, Russia; Smirnov_Michael@mail.ru (M.A.S); alexander.n.bugrov@gmail.com (A.N.B.); a.toikka@spbu.ru (A.M.T.); 2 Lappeenranta University of Technology, Laboratory of Physics, Skinnarilankatu 34, Lappeenranta 53850, Finland; Pavel.Geydt@lut.fi (P.G.); Erkki.Lahderanta@lut.fi (E.L.); 3 Lappeenranta University of Technology, Group of Packaging Technology, Skinnarilankatu 34, Lappeenranta 53850, Finland; sovaska@lut.fi; 4 Institute of Macromolecular Compounds, Russian Academy of Sciences, Bolshoy pr. 31, Saint Petersburg 199004, Russia

**Keywords:** polyimide, zirconia, mixed matrix membrane, polymer structure, Quantitative Nanomechanical Mapping, pervaporation

## Abstract

Mixed-matrix membranes based on amorphous and semi-crystalline polyimides with zirconium dioxide (ZrO_2_) nanostars were synthesized. Amorphous poly(4,4′-oxydiphenylenepyromellitimide) and semi-crystalline polyimide prepared from 1,4-bis(4-aminophenoxy)benzene and 4,4’-oxydiphthalic anhydride were used. The effect of ZrO_2_ nanostars on the structure and morphology of nanocomposite membranes was studied by wide-angle X-ray scattering, scanning electron microscopy, atomic force microscopy, and contact angle measurements. Thermal properties and stability were investigated by thermogravimetric analysis and differential scanning calorimetry. Transport properties of hybrid membranes containing 5 wt % ZrO_2_ were tested for pervaporation of a mixture of butanol–water with 10 wt % H_2_O content. It was found that a significant amount of the ZrO_2_ added to the semi-crystalline polyimide is encapsulated inside spherulites. Therefore, the beneficial influence of inorganic filler on the selectivity of mixed-matrix membrane with respect to water was hampered. Mixed-matrix membranes based on amorphous polymer demonstrated the best performance, because water molecules had higher access to inorganic particles.

## 1. Introduction

Aromatic heterocyclic polyimides and their composites are promising membrane materials [1,2,3,4,5] due to their superior chemical stability, good mechanical properties, and excellent thermal stability. The effectiveness of most membrane processes (such as pervaporation) is mainly controlled by the selection of membrane material with appropriate structural, physical, and chemical properties [6]. At the same time, inorganic membranes demonstrate better separation performance compared to polymeric ones [7]. Therefore, exactly composite organic–inorganic or so-called mixed-matrix membranes (MMMs) that combine the advantages of both types of materials are widely investigated [8,9]. The packing of polymeric chains inside the membrane and the amount of free volume are main factors regulating the diffusion of the penetrants through the membrane [10,11]. The introduction of inorganic nanoparticles into an organic membrane can: (1) decrease chain mobility near polymer–particle interface [12]; (2) change the membrane’s free volume [13,14]; and (3) change both the degree of crystallinity and the sizes of polymer spherulites [11]. All of these factors affect the permeability of the membrane. The appearance of new water-selective diffusion pathways along polymer–inorganic interface should increase the selectivity of a membrane. However, the level of such an increase may be reduced by the blockage of selective channels because of the crystalline nature of the polymer and/or a decrease of chain mobility near polymer–particle interface. For better understanding of structure–properties correlation in MMMs based on polyimides, it is interesting to compare the impact of the introduction of inorganic nanoparticles into the amorphous and semi-crystalline polymers. The influence of polyimide crystallinity on the structure of MMMs has not yet been studied. This was the motivation to investigate two polyimides in this work. The first polyimide was amorphous poly(4,4′-oxydiphenylenepyromellitimide) (PMDA-ODA), and the second was a semi-crystalline polyimide based on 1,4-bis(4-aminophenoxy)benzene with 4,4’-oxydiphthalic anhydride (TPEQ-ODPA).

Nanoparticles of zirconium dioxide (ZrO_2_) synthesized via the hydrothermal method can be obtained in different shapes [15,16,17]. They can operate as inorganic fillers, which regulate the physico-chemical properties of polymer materials [18,19]. According to the previous results of Bugrov et al. [17,20] and data presented in the literature [21,22,23], two main advantages of using ZrO_2_ nanostars over other ZrO_2_ nanofiller shapes can be specified in the case of MMMs for alcohol dehydration via pervaporation. First, nanostars demonstrate higher specific surface in comparison with ZrO_2_ in the form of spheres (87 m^2^·g^−1^) and rods (17 m^2^·g^−1^). The specific surface of nanostars (121 m^2^·g^−1^) is slightly lower than the value for ZrO_2_ hollow spheres (140 m^2^·g^−1^), but hollow spheres are characterized by a wide distribution of dimensions (300–700 nm) and indicate low mechanical stability. Secondly, in the case of nanostars, the particle surface has the highest concentration of polar groups due to the synthetic procedure [23]. Summarizing both factors, it can be concluded that nanostars contains the highest amount of specific sorption centers that are beneficial for selective interaction with water molecules. Unfortunately, the literature possesses no information about the influence of such nanoparticles on the structure and transport properties of polyimide membranes. This is why we chose ZrO_2_ nanostars as the inorganic filler for the preparation of MMMs in this work.

The transport properties of the obtained nanocomposite material were studied in the pervaporation of a *n*-butanol–water mixture. We consider this constituent of research as a testing of the MMMs material. On the other hand, *n*-butanol dehydration has a well-known practical significance. *n*-Butanol is a bulk chemical that is now widely considered as a direct replacement of gasoline or as a fuel additive [24,25,26]. Industrial production of *n*-butanol is realized via either petrochemical or biobased processes. The latter fabrication process has essential importance as a method to produce fuel from renewable resources [24,27,28]. Increasing the usage and production of *n*-butanol in the biochemical industries determined the publication of a number of recent works concerning pervaporative separation of *n*-butanol from aqueous solutions (e.g., [29,30,31,32,33,34]). Distillation and adsorption remain dominant industrial process for the enrichment of bio-butanol. However, since they are energy intensive, high production cost limits the further promotion of such biofuels [35]. The importance of *n*-butanol was the reason for the choice the *n*-butanol–water mixture as a model mixture in our study.

## 2. Materials and Methods

### 2.1. Materials

Zirconium(IV) oxychloride octahydrate (98.5%, Neva-Reactive, Saint Petersburg, Russia, CAS: 7699-43-6); sodium acetate trihydrate (99.5%, Neva-Reactive, Saint Petersburg, Russia, CAS: 6131-90-4); *N*-methyl-2-pyrrolidone (97%, Sigma-Aldrich, St. Louis, MO, USA, CAS: 120-94-5); 4,4′-oxydiphthalic anhydride (>98.0%, TCI, CAS: 1823-59-2); 1,4-bis(4-aminophenoxy)benzene (>98.0%, TCI, CAS: 3491-12-1); pyromellitic dianhydrid (99.0%, TCI, CAS: 89-32-7), and 4,4’-diaminodiphenyl ether (>98.0%, TCI, CAS: 101-80-4) were used as received without purification. Butanol for the preparation of aqueous-alcohol mixture was purchased from Vekton (Saint Petersburg, Russia).

### 2.2. Synthesis of ZrO_2_ Nanostars

Star-shaped ZrO_2_ nanoparticles were synthesized in hydrothermal conditions from ZrOCl_2_·8H_2_O, by the method described previously in [23]. Zirconium oxychloride (0.805 g) and sodium acetate (0.103 g)—i.e., their mole ratio was 1:2—were dissolved in 15 mL of distilled water under stirring for 1 h. The obtained solution was transferred to a teflon cell and was kept in an autoclave at a temperature of 240 °C and pressure of 150 atm for 4 h. The powder obtained after hydrothermal synthesis was washed with distilled water, dried, and then dispersed in *N*-methyl-2-pyrrolidone (NMP) under the ultrasonic treatment.

### 2.3. Preparation of MMMs

**Figure 1:**
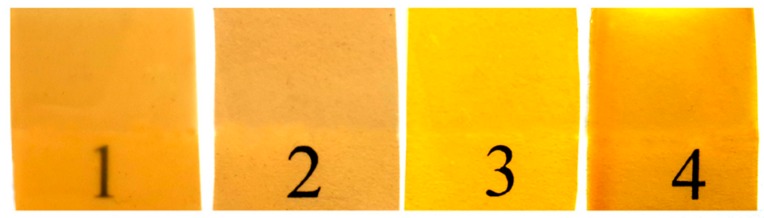
Optical images of membranes: (**1**) TPEQ-ODPA (polyimide based on 1,4-bis(4-aminophenoxy)benzene with 4,4’-oxydiphthalic anhydride); (**2**) Prepared mixed-matrix membrane (MMM) with TPEQ-ODPA (TPEQ-ODPA-S); (**3**) PMDA-ODA (poly(4,4′-oxydiphenylenepyromellitimide)); and (**4**) prepared MMM with PMDA-ODA (PMDA-ODA-S).

For the preparation of polyimides, two sets of monomers were used: (1) 1,4-bis(aminophenoxy)benzene and 4,4′-oxydiphthalic anhydride for TPEQ-ODPA; (2) pyromellitic dianhydride and 4,4’-diaminodiphenyl ether for PMDA-ODA. ZrO_2_ nanostars with concentration 5 wt % based on the weight of a polymer were dispersed in NMP. Then, the corresponding diamines and dianhydrides in a molar ratio of 1:1.03 were consistently dissolved in the dispersion of nanoparticles. The formation of polyamic acid was conducted under argon flow during 6 h. Membranes were prepared by solution casting method with subsequent drying for 12 h at 80 °C. Imidization reaction was performed by step-wise rising of annealing temperature during the process: 1 h at 100 °C, 1 h at 150 °C, 1 h at 200 °C, 1 h at 250 °C, 1 h at 280 °C, and 0.5 h at 300 °C. Finally, obtained membranes were removed from glass plates for further investigation. Prepared MMMs are denoted in the text as TPEQ-ODPA-S and PMDA-ODA-S. For comparison, the same membranes without the addition of nanostars were prepared. They are denoted as TPEQ-ODPA and PMDA-ODA, respectively. Optical images of membranes are shown in Figure 1. The thickness of all membranes was 20 µm.

### 2.4. Characterization Methods

#### 2.4.1. Microscopic Investigation

ZrO_2_ nanostar powder was dispersed in ethanol using an ultrasonic bath. Grids with graphene oxide films prepared by the method elaborated in [36] were drenched in the ZrO_2_ suspension and dried. After that, the samples with ZrO_2_ nanostars were investigated using a Philips EM420 (Philips Electron Optics, Eindhoven, The Netherlands) transmission electron microscope (TEM) at an accelerating voltage of 80 kV. Bright field image and electron diffraction patterns were obtained.

Scanning electron microscopy (SEM) micrographs were obtained with a Zeiss Merlin SEM (Carl Zeiss, Oberkochen, Germany) at 5 kV voltage. For investigation of cross-sections, the membranes were submerged in liquid nitrogen and fractured perpendicularly to their surface.

Scanning Probe Microscope Multimode 8 (Bruker, Santa Barbara, CA, USA) operating in PeakForce TUNA^TM^ mode was used for atomic force microscopy (AFM) experiments. Scanning was done in PeakForce calibrated Quantitative Nanomechanical Mapping (QNM) mode with feedback adjusted automatically by ScanAsyst program protocol. PeakForce parameters were: amplitude 100 nm and frequency 2 kHz. ScanAsyst-Air probe (Bruker, Santa Barbara, CA, USA) with tip radius 5 nm and spring constant 0.47 N·m^−1^ was used for accurate topography measurements with setpoint force 2 nN. Then, considerably stiffer (correspondingly: 10 nm, 120 N·m^−1^, and resonance frequency 447 kHz) Tap525a probe (Bruker, Santa Barbara, CA, USA) was utilized to carry out the QNM measurements under a force of ~50 nN that allowed the sample to be deformed by approximately 1 nm in depth. The calibration of the stiff cantilever probe in this work was done in accordance with guidelines declared by Sader in [37].

#### 2.4.2. Wide-Angle X-Ray Diffraction Study

Wide angle X-ray diffraction (WAXD) analysis of initial polyimides and MMMs were obtained at room temperature using a D8 DISCOVER diffractometer (Bruker, Rheinstetten, Germany) at scattering angles varying from 5° to 60° with 0.05° step. Cu-Kα radiation (40 kV, 40 mA) was used. The volume fraction of crystalline regions (χ, %) was calculated according to the equation:

(1) $$\chi =\frac{\int_{0}^{\infty }I_{\mathrm{cr}}\left(q\right)dq}{\int_{0}^{\infty }\left[I_{\mathrm{cr}}\left(q\right)+I_{\mathrm{am}}\left(q\right)\right]dq},$$

where *I*_cr_ and *I*_am_ are intensities that arise from diffraction on the crystalline and amorphous regions, and *q* is the length of the scattering vector. Deconvolution of WAXD curves was performed before calculation of degree of polymer crystallinity in order to exclude additional crystallinity arising from ZrO_2_ nanostars.

The identification of crystalline phase in the ZrO_2_ was performed by comparing our data with the Joint Committee for Powder Diffraction Standards (JCPDS) files. The Bruker Topas 4.2 software was used for calculation of lattice parameters from WAXD data.

#### 2.4.3. Analysis of Thermal Properties

Differential scanning calorimetry (DSC) was conducted using a DSC 204 F1 (Netzsch, Selb, Germany) differential scanning calorimeter to assess the glass transition temperature (*T*_g_) of samples. The analysis was conducted under inert atmosphere with samples of approximately 4–5 mg at a scan rate of 20 °C·min^−1^ from 20 to 350 °C. Thermobalance TG 209 F1 Libra (Netzsch, Selb, Germany) was used for thermogravimetric analysis (TGA), which was performed under inert atmosphere with samples having a weight of approximately 2–4 mg at a scan rate of 10 °C·min^−1^ from 40 to 950 °C.

#### 2.4.4. Contact Angle Measurements

Apparent contact angles were measured under ambient conditions (23 °C, 50% RH) with a Theta optical tensiometer (Biolin Scientific AB, Stockholm, Sweden) using deionised water as a test liquid. A drop with a volume of 3 µL was injected on the membrane’s surface using an automatic single liquid dispensing system with a 200 µL tip. A 420 Hz camera (Basler A602F-2 with Navitar Inc., Rochester, NY, USA) was used to capture images of the drop placed on each membrane. Digital images were analyzed with the OneAttension image tool software. The contact angle value was read 1 s after dispensing the drop.

#### 2.4.5. Pervaporation Experiments

A laboratory pervaporation system was built with an effective membrane area of 2.6 cm^2^ working at 40 °C (this temperature is typical for pervaporation of a butanol–water system in the literature; e.g., [38,39]). Downstream pressure was kept at less than 0.1 mbar by applying a vacuum pump. Permeate was collected in a cold trap immersed in liquid nitrogen and containing a Dewar flask to condense vapor to the liquid phase, weighed, and then analyzed with gas chromatograph “Chromatec Crystal 5000.2” (Chromatec Company, Yoshkar-Ola, Russia) with a thermal conductivity detector.

The fluxes, *J* (kg·m^−2^·h^−1^), were determined as the amount of liquid transported through a unit of the membrane area per hour. Selectivity (α) of the membranes respective to water was calculated as α = (*x*_p_*y_f_)/(*x*_f_*y_p_), where *x*_f_, *y*_f_, *x*_p_, and *y*_p_ are the mass fractions of water (*x*) and butanol (*y*) in feed (index *f*) and permeate (index *p*) mixtures, respectively.

#### 2.4.6. Mechanical Measurements

Mechanical properties of the initial polyimides and MMMs on their base were measured in tensile mode (speed 5 mm·min^−1^) with Instron Mikro Tester 5940 (Norwood, MA, USA) mechanical testing machine. Membranes were cut in stripes with sizes 20 × 2 mm^2^ for this investigation.

## 3. Results and Discussion

### 3.1. Investigation of Morphology with Electron Microscopy

**Figure 2:**
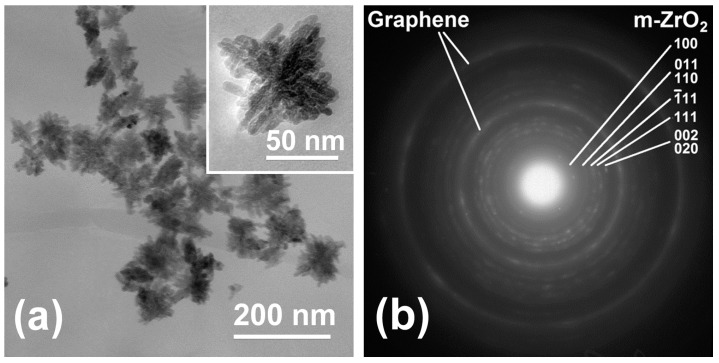
Transmission electron microscopy (TEM) image of (**a**) zirconia nanostars, and (**b**) their electron diffraction picture.

The TEM micrograph of pure zirconia nanostars prepared by hydrothermal procedure is shown in Figure 2a. It is seen that diameter of zirconia stars is in the range of 50–100 nm and the thickness of beams is 7–12 nm (see image inserted in Figure 2a). The electron diffraction picture (presented in Figure 2b) gives evidence that the zirconia synthesized by hydrothermal procedure in our case have monoclinic crystalline structure.

**Figure 3:**
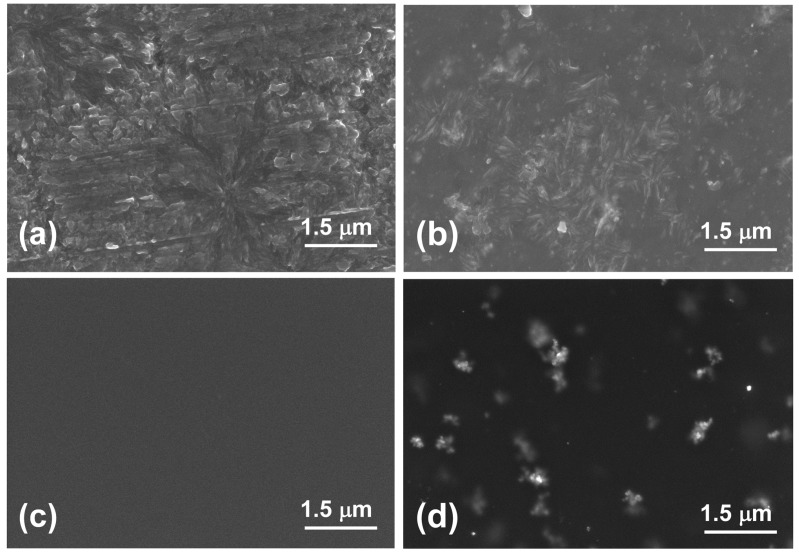
Scanning electron microscopy (SEM) images of the surface of (**a**) TPEQ-ODPA; (**b**) TPEQ-ODPA-S; (**c**) PMDA-ODA; and (**d**) PMDA-ODA-S.

**Figure 4:**
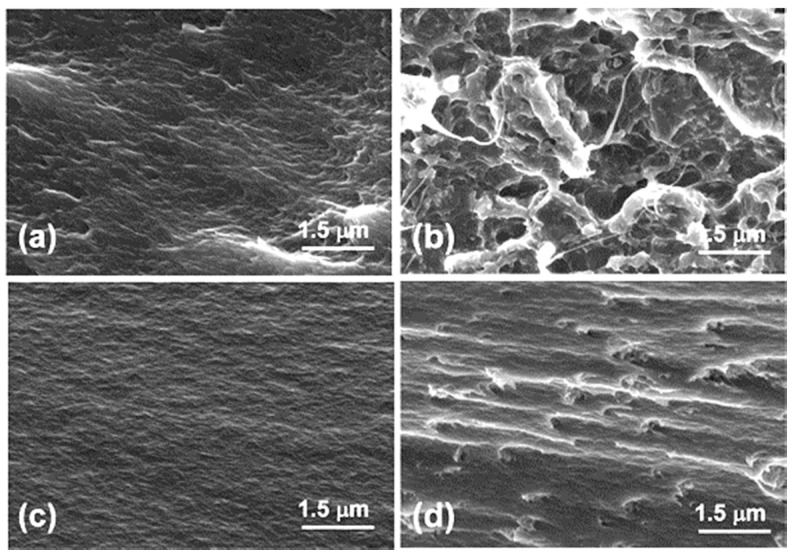
Cross-section morphology of (**a**) TPEQ-ODPA; (**b**) TPEQ-ODPA-S; (**c**) PMDA-ODA; and (**d**) PMDA-ODA-S.

Figure 3 and Figure 4 show the SEM images of the surface and the cross-sections of polyimide membranes and MMMs. Large spherulites with size of ~7 μm are visible on the surface of TPEQ-ODPA (Figure 3a). At the same time, the surface of the PMDA-ODA membrane (Figure 3c) is smooth. The introduction of 5% of zirconia nanostars led to a significant change of the morphology of TPEQ-ODPA. The size of the spherulites decreased to 1 μm (Figure 3b). This may be explained by the ability of nanostars to be nucleation centers for the formation of crystallites during membrane preparation [40,41], which increases the number and decreases the size of spherulites. Uniform distribution of nanostars and their clusters on the surface of MMMs is seen in images for both membranes (Figure 3b,d). This is more clearly seen for the PMDA-ODA-S membrane (Figure 3d) due to smooth morphology of the corresponding matrix (Figure 3c). The sizes of inorganic inclusions that can be measured from SEM pictures of surface are 80–500 nm. Agglomeration of the nanostars on the surface of membrane can be explained by the fact that particles which are deposited near the surface or on the surface have a lack of contact with polymer. Nanostars have the tendency to agglomerate because of their high surface area and surface tension. Sizes of inorganic inclusions (as can be estimated from cross-section) lie in the range 80–200 nm. Due to the strong interaction between functional groups in the polymer and the surface of the inorganic particles, ZrO_2_ nanostars probably form the mesh nodes of physical links, playing the role of intermolecular cross-linker [42,43], which prevents the increasing degree of nanofiller agglomeration inside the volume of the membrane.

Dense packing structure was observed in cross-section SEM images of initial polyimide membranes (Figure 4a,c). A more curved cross-section of the TPEQ-ODPA matrix (Figure 4a) can be connected to presence of spherulites in this membrane, and/or with different chain flexibility of the investigated polyimides. A dramatic difference is clearly seen between the view of MMM cross-sections (Figure 4b,d) and the corresponding polymeric matrices. Cavities inside the membrane are observed for TPEQ-ODPA-S (Figure 4b). A possible mechanism of their formation may be proposed on the basis of the thermal properties of the TPEQ-ODPA matrix polymer, which will be discussed further. In the case of PMDA-ODA-S mixed-matrix membrane (see Figure 4d), multiple fracture lines are seen (which is not the case for pure PMDA-ODA matrix Figure 4c), and is associated with presence of inorganic particles in the membrane’s volume. ZrO_2_ nanostars and their clusters are observed in the beginning of almost each fracture line (Figure 4d). The common problem in the preparation of MMMs is the lack of interface compatibility between organic polymer and inorganic filler, and nonuniform particle distribution inside membrane [44]. Figure 4d gives evidence that, in our case, inorganic particles have low tendency to agglomerate in the bulk of the membrane.

### 3.2. AFM Results

**Figure 5:**
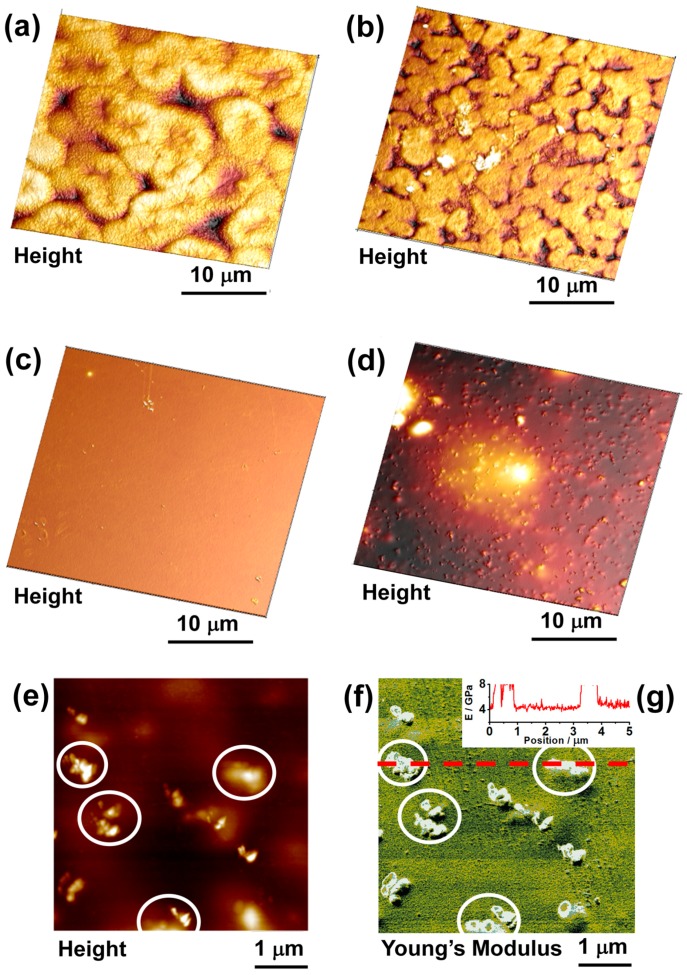
3D models of surface topography of (**a**) TPEQ-ODPA; (**b**) TPEQ-ODPA-S; (**c**) PMDA-ODA; and (**d**) PMDA-ODA-S obtained from AFM measurements. (**e**) Detailed topography and (**f**) map of Young’s modulus for the same area of the surface of the PMDA-ODA-S mixed matrix membrane. (**g**) Profile of the Young’s modulus data for PDMA-ODA-S at the location indicated with a dotted line in (**f**). Color schemes for images (**a**–**e**) were adjusted to highlight morphological features of each sample, while equal dimensionality by *X*-*Y*-*Z* axes provides a realistic representation of the surfaces.

Results of the AFM investigation of pure polyimide membranes and MMMs are presented in Figure 5. 3D-topographies of TPEQ-ODPA (Figure 5a) and TPEQ-ODPA-S (Figure 5b) membranes clearly demonstrate the shape of spherulites and the change in shape upon the introduction of ZrO_2_ particles into the polymer matrix. The initial polymer membrane (Figure 5a) is characterized by considerable variation of spherulite sizes from 4 to 10 μm, while for MMM, this parameter lies in the range 1–4 μm. This observation is in agreement with our SEM results, which are presented in Section 3.1. The surface of the PMDA-ODA membrane demonstrates smooth morphology (Figure 5c). However, the introduction of nanostars into PMDA-ODA leads to the emergence of uniformly-distributed hills (Figure 5d) with sizes resembling those of clusters of ZrO_2_ filler (see Section 3.1).

The smooth morphology of PMDA-ODA-S (Figure 5f) made it possible to investigate the elastic characteristics of this membrane by AFM operating in PeakForce QNM mode. This advanced AFM technique allows the mapping of certain mechanical properties simultaneously with topography and with the same spatial resolution. This approach was recently shown to be a powerful tool in the characterization of both structure and local mechanical properties of hybrid materials at the nanoscale [45]. The results presented in Figure 5e,f show topography and mapping of the Young’s modulus of the same area of the PMDA-ODA-S membrane. Figure 5g demonstrates a profile line over the map of elastic modulus on Figure 5f. Its baseline corresponds to the polymer phase, while sharp peaks indicate excessively rigid ZrO_2_ inclusions. According to obtained elastic maps, the Young’s modulus of the PMDA-ODA-S mixed-matrix membrane polymeric matrix was approximately 4 GPa. This value resembles the one that was previously reported in the literature for PMDA-ODA [46]. Exact measurement of the Young’s modulus of inorganic inclusions was not possible, because the rigidity value of ZrO_2_ is significantly higher than the upper limit of the utilized AFM probe. Nevertheless, inorganic nanostars can be observed with considerable color contrast, as seen in Figure 5f. Comparison between Figure 5e,f provides evidence that the hills that are visible in topography correspond to the rigid inorganic particles that are fixed on top of the polymeric surface (specific representative parts of the surface are marked with circles).

### 3.3. WAXD Data

**Figure 6:**
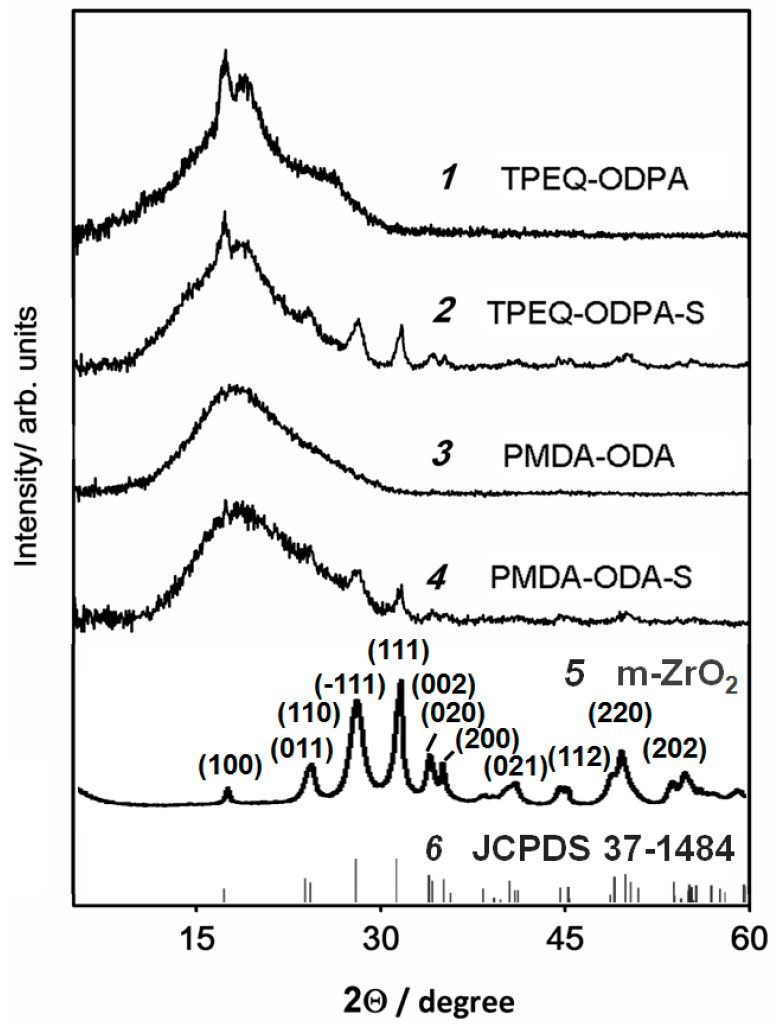
Wide angle X-ray diffraction (WAXD) patterns for the (**1**) TPEQ-ODPA; (**2**) TPEQ-ODPA-S; (**3**) PMDA-ODA; (**4**) PMDA-ODA-S; (**5**) ZrO_2_; and (**6**) reference pattern for monoclinic zirconia.

WAXD patterns of the polyimide membranes (obtained in reflection modes) are shown in Figure 6, pattern 1–4. It is seen that membrane TPEQ-ODPA (Figure 6, pattern 1) is semi-crystalline. Sample exhibited X-ray diffraction patterns with strong reflections at 17.4° and 18.9° with spacings *d* = 5.1 and 4.7 Å, respectively, and weak reflection at 25.5° (*d* = 3.5 Å). The degree of crystallinity of TPEQ-ODPA χ = 28% was calculated with Equation (1). The WAXD pattern of sample TPEQ-ODPA-S (Figure 5, pattern 2) exhibited main reflections at nominally identical locations. However, the intensity of the reflections at 17.4° and 18.9° of MMM (Figure 5, pattern 2) are slightly lower than that for pure polymer. This means a lower degree of crystallinity of MMM than for the initial polymeric matrix. In addition, the reflection at 25.5° disappeared. The degree of crystallinity of TPEQ-ODPA-S, as it can be calculated from the WAXD pattern, is 16%. Reducing the degree of crystallinity is apparently associated with the number of crystallization nuclei and the formation of a finer spherulitic structure [47]. At the same time, strong reflections with 2θ = 24.1°, 28.1°, 31.5°, 34.2°, 35.2°, 44.7°, 50.0°, and 54.2° (*d* = 3.7, 3.2, 2.8, 2.6, 2.5, 1.9, 1.8, and 1.7 Å) appear, which are in the same position as for initial ZrO_2_ nanostars (Figure 6, pattern 5). The WAXD pattern of ZrO_2_ nanostars have a number of well resolved peaks at 2θ = 17.2°, 24.1°, 28.1°, 31.5°, 34.2°, 35.2°, 44.7°, 50.0°, and 54.2°. These peaks coincide with JCPDS card No. 37-1484 (Figure 6, pattern 6) for monoclinic zirconia, and indexed as reflection from (100), (011, 100), (−111), (111), (002, 020), (200), (112), (220), and (202) planes, respectively. Additionally, lattice parameters were calculated with Bruker Topas 4.2 software. Obtained values are *a* = 5.18, *b* = 5.23, *c* = 5.35, and β = 99.7°. Lattice parameters reveal the presence of a monoclinic ZrO_2_ crystal phase in MMMs that is in agreement with our TEM results (Figure 2b).

As can be seen in Figure 6, patterns 3 and 4, the PMDA-ODA matrix and PMDA-ODA-S have a broad peak in the X-ray diffraction patterns at 18.2° (*d* = 4.9 Å) that reveals their amorphous structure. After the incorporation of inorganic particles into the PMDA-ODA, reflections which correspond to the ZrO_2_ phase appear at the same position (Figure 6, pattern 4) as they were observed for TPEQ-ODPA-S.

### 3.4. Thermal Stability

**Figure 7:**
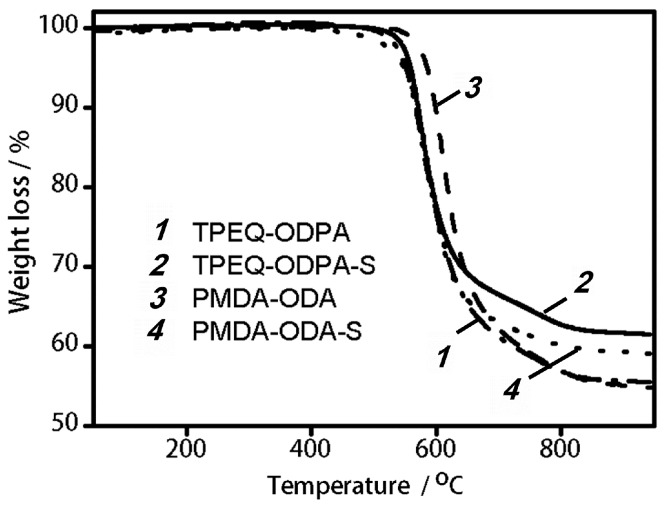
Thermogravimetric analysis (TGA) curves for the TPEQ-ODPA, TPEQ-ODPA-S, PMDA-ODA, and PMDA-ODA-S.

Thermal stability was determined by TGA (Figure 7). The region of major weight loss is the same for all investigated samples. It lies in the range of 500–800 °C, and corresponds to decomposition of the polymer backbone. At the same time, the impact of ZrO_2_ on decomposition temperature depends on the matrix polymer. This temperature is approximately 490 °C for TPEQ-ODPA and TPEQ-ODPA-S. However, in the case of PMDA-ODA and PMDA-ODA-S, the mass loss starts at 450 °C for MMMs and at 550 °C for pure matrix polymer. This may be associated with lower molecular mass of polymer chains in PMDA-ODA-S that is synthesized in the presence of zirconia nanostars in comparison with the weight of polymer chains in pure PMDA-ODA. Indeed, ZrO_2_ is able to interact with dianhydrides in dispersion before polymerization, as was observed for ZnO in [43]. The total weight loss achieved at 900 °C is lower for MMMs (Figure 7, curves 2 and 4) in comparison with pure polymeric matrices (Figure 7, curves 1 and 3). This is connected with presence of thermally stable ZrO_2_. Thus, significant thermal stability of both pure matrices and prepared MMMs was proved.

DSC investigations were carried out in the temperature range 20–350 °C. The glass transition temperatures (*T*_g_) for TPEQ-ODPA and TPEQ-ODPA-S were found to be 237 and 203 °C, respectively. The reason for the decrease in *T*_g_ is the lower degree of crystallinity of TPEQ-ODPA-S in comparison with initial matrix polymer, as was discussed earlier (Section 3.3). The glass transition temperature was not determined for membranes based on PMDA-ODA. This means that *T*_g_ for these systems is higher than 350 °C, which is in agreement with the literature [48]. Melting temperature was achieved only in the case of TPEQ-ODPA-S, and the values of *T*_m_ = 316 °C and Δ*H* = 23.3 J·g^−1^ were measured. For other investigated materials, the value of T_m_ is higher than 350 °C [49].

### 3.5. Measurements of Contact Angle with Water

**Figure 8:**
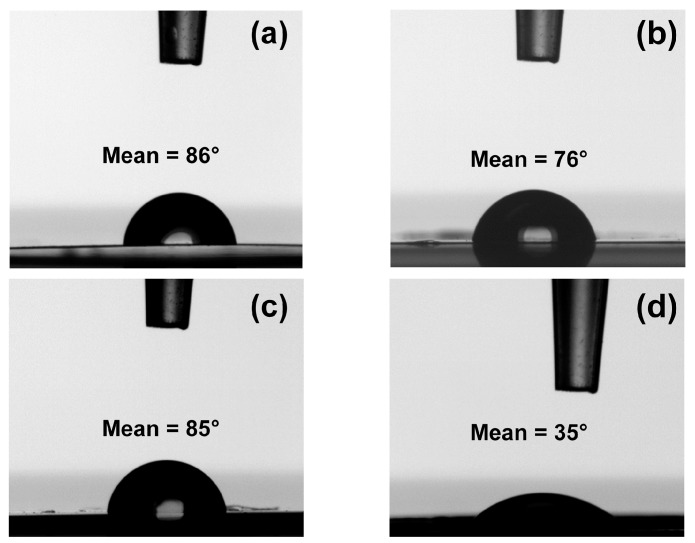
Water droplets on the surface of (**a**) TPEQ-ODPA; (**b**) TPEQ-ODPA-S; (**c**) PMDA-ODA; and (**d**) PMDA-ODA-S after 1 s contact time.

The contact angle experiments were conducted for better understanding of the impact of ZrO_2_ nanostars on the surface properties of polyimide membranes. A series of optical images of water droplets on the surface of membranes are presented in Figure 8. It is seen that both initial polyimide membranes are slightly hydrophilic (Figure 8a,c), since the registered values of contact angle were slightly below 90° (i.e., 86° for TPEQ-ODPA and 85° for PMDA-ODA). The addition of inorganic filler led to an increase of the hydrophilicity of both polyimides. Contact angles 76° and 35° were obtained for TPEQ-ODPA-S and PMDA-ODA-S, respectively (Figure 8b,d). The angles showed only a minor decrease during the 10 s test period, and the volume of the drop was also relatively stable, suggesting that only moderate liquid adsorption and spreading took place. The contact angle results are in good agreement with the measurements of transport properties of initial and mixed-matrix membranes in the pervaporation experiment. The more hydrophilic membrane (PMDA-ODA-S) demonstrated higher selectivity with respect to water, which will be discussed later. The decrease of the contact angle is more significant for membranes based on PMDA-ODA then for TPEQ-ODPA systems (Figure 8b,d). This can be explained as follows. In the TPEQ-ODPA-S membrane, the ZrO_2_ nanoparticles act as crystallization centers, so many of them are captured inside the crystalline part of the polymer. At the same time, ZrO_2_ in the amorphous PMDA-ODA matrix has a higher possibility to come near the surface because the crystalline part does not exist in this case.

### 3.6. Pervaporation Experiments

In our work, the butanol–water (10 wt % of H_2_O) system was used to test the impact of ZrO_2_ nanostars on the transport properties of a polyimide membrane. The results are presented in Table 1. It can be seen that addition of ZrO_2_ nanostars led to an increase in selectivity for both types of membranes. The increase in selectivity with respect to water is connected to the hydrophilic character of ZrO_2_, which provides the formation of water-selective channels along the organic–inorganic interface. Higher selectivity of PMDA-ODA-S in comparison with TPEQ-ODPA-S is explained by higher wettability and better access of penetrants to the ZrO_2_–polymer interface inside the PMDA-ODA-S membrane. The presence of cavities was found in the SEM investigation of the TPEQ-ODPA-S membrane cross-section, which was discussed earlier (Figure 4b). The formation of cavities only in TPEQ-ODPA-S can be explained by following considerations. The final stage of thermal imidization of polyimide membranes was conducted at 300 °C (i.e., higher than the glass-transition temperature for TPEQ-ODPA-S). Moreover, only for this sample, the temperature of thermal imidization comes near its melting temperature. At the same time, these heating conditions are far from the glass transition temperature of PMDA-ODA. Thus, the mobility of polymer chains during the final stage of imidization at 300 °C is the highest for the TPEQ-ODPA-S membrane. The maximum probability of the formation of interchain cross-links via the interaction of polyamic acid and –OH groups on the surface of ZrO_2_ nanoparticles can thus be expected. As a result, the degree of polymer crystallinity decreases, and inhomogeneity of the inner membrane structure can increase. All of these considered factors lead to the formation of the cavities that cause the increase of permeability of the TPEQ-ODPA-S membrane in comparison with pure TPEQ-ODPA (See Table 1). A decrease in flux with the addition of ZrO_2_ nanostars to the PMDA-ODA matrix is presumed to be connected with possible cross-linking by the interaction of surface –OH groups of the inorganic phase and –COOH groups of the polymer before imidization. This possibility is confirmed by the mechanical measurements of Young’s modulus of samples in tensile mode. It was observed that the introduction of ZrO_2_ nanostars led to an increase of the Young’s modulus from 2.0 to 2.8 GPa for amorphous polyimide, and from 2.9 to 3.1 GPa for semi-crystalline.

## 4. Conclusions

Novel MMMs based on polyimides and ZrO_2_ nanostars were successfully prepared and investigated. The uniform distribution of ZrO_2_ nanostars in the membrane volume was achieved due to the unique preparation method, which includes dispersion of inorganic particles in the solution of monomers with subsequent polymerization and thermal imidization. The influence of polyimides crystallinity on the structure and transport properties of MMMs was investigated for the first time. It was shown that in situ introduction of ZrO_2_ nanostars at the stage of formation of prepolymer (polyamic acid) led to a uniform distribution of filler in the membrane’s volume. Pervaporation measurements revealed that the addition of inorganic nanoparticles led to an increase of selectivity for both polyimide membranes with respect to water due to the hydrophilic nature of the inclusions. The mentioned effect was more pronounced for amorphous polyimide, which was explained on the basis of structural investigations by better access of water molecules to the inorganic particles on the surface and inside the volume of this polymer. The addition of zirconia led to an increase of flux though the semi-crystalline polyimide due to a decrease of its degree of crystallinity and the formation of cavities. At the same time, the diffusion rate of penetrants through the amorphous polymer decreased. This can be explained by the cross-linking activity of ZrO_2_ nanostars. However, total flux through MMMs based on amorphous polyimide was still higher than flux measured for membranes prepared with semi-crystalline polymer (0.14 and 0.13 kg·m^−2^·h^−1^, respectively). Thus, MMMs based on exactly amorphous polyimides with ZrO_2_ nanostars can be proposed as promising membrane materials.

## Figures and Tables

**Table 1:** Pervaporation of butanol–water mixture (10 wt % of water) using polyimide membranes and MMMs.

Membrane	H_2_O content in permeate, wt %	Selectivity	Flux, kg·m^−2^·h^−1^
TPEQ-ODPA	28	3.4	0.076
TPEQ-ODPA-S	65	17.0	0.130
PMDA-ODA	26	3.2	0.235
PMDA-ODA-S	92	109.3	0.140
